# Replicative Stress Coincides with Impaired Nuclear DNA Damage Response in COX4-1 Deficiency

**DOI:** 10.3390/ijms23084149

**Published:** 2022-04-08

**Authors:** Liza Douiev, Chaya Miller, Guy Keller, Hadar Benyamini, Bassam Abu-Libdeh, Ann Saada

**Affiliations:** 1 Department of Genetics, Hadassah Medical Center, Jerusalem 9112001, Israel; chaya.miller7@gmail.com (C.M.); guy.keller@mail.huji.ac.il (G.K.); 2 Info-CORE, Bioinformatics Unit of the I-CORE, Hebrew University of Jerusalem, Jerusalem 9112001, Israel; hadar.benyamini@gmail.com; 3 Department of Pediatrics and Genetics, Makassed Hospital and Al-Quds University, East Jerusalem, Palestinian Territories 91220, Israel; drbassam@staff.alquds.edu; 4 Faculty of Medicine, Hebrew University of Jerusalem, Jerusalem 9112001, Israel

**Keywords:** *COX4i1*, cytochrome c oxidase, mitochondria, mitochondrial respiratory chain, replicative stress, DNA damage

## Abstract

Cytochrome *c* oxidase (COX), a multimeric protein complex, is the final electron acceptor in the mitochondrial electron transfer chain. Primary COX deficiency, caused by mutations in either mitochondrial DNA or nuclear-encoded genes, is a heterogenous group of mitochondrial diseases with a wide range of presentations, ranging from fatal infantile to subtler. We previously reported a patient with primary COX deficiency due to a pathogenic variant in *COX4I1* (encoding the common isoform of COX subunit 4, COX4-1), who presented with bone marrow failure, genomic instability, and short stature, mimicking Fanconi anemia (FA). In the present study, we demonstrated that accumulative DNA damage coincided primarily with proliferative cells in the patient’s fibroblasts and in *COX4i1* knockdown cells. Expression analysis implicated a reduction in DNA damage response pathways, which was verified by demonstrating impaired recovery from genotoxic insult and decreased DNA repair. The premature senescence of the COX4-1-deficient cells prevented us from undertaking additional studies; nevertheless, taken together, our results indicate replicative stress and impaired nuclear DNA damage response in COX4-1 deficiency. Interestingly, our in vitro findings recapitulated the patient’s presentation and present status.

## 1. Introduction

The mitochondrion is an intracellular cytosolic double-membrane-bound organelle. It plays a critical role in many cellular processes, and its most important role is the generation of energy as adenosine triphosphate (ATP) via the oxidative phosphorylation system (OXPHOS). This system is localized in the inner mitochondrial membrane and is organized into five multimeric protein complexes (CI–CV) and cytochrome c and coenzyme Q. Electrons derived from nutrients are transferred via complexes I–IV (the electron transfer chain, ECT), while three of these are proton pumps (CI, CIII, and CIV), generating an electrochemical gradient, which is utilized by CV (ATP synthase) to generate ATP. CIV (cytochrome c oxidase, COX) is the terminal electron acceptor, catalyzing the reduction of molecular oxygen to form water. Mammalian COX is a dimeric complex composed of 14 subunits, of which the largest three (COX I, II, III) are encoded by the mitochondrial genome, while the remaining are nuclear-encoded. The synthesis and assembly of COX depends on numerous assembly proteins, while its activity is regulated by allosteric and post-translational mechanisms and by the expression of tissue-specific isoforms [1,2,3].

Primary COX deficiency belongs to a subgroup of mitochondrial diseases, and up to 30 pathogenic variants related to human mitochondrial COX deficiency have been reported. The clinical manifestations of primary COX deficiencies are heterogeneous, ranging from fatal infantile to subtler, mainly involving the brain and muscle, but frequently also the heart, liver, and pancreas [4,5,6]. Additionally, we and others have reported the occurrence of hematopoietic system involvement in patients with isolated COX deficiency [4,5,6,7,8,9]. In 2017, we reported a pathogenic variant (K101N) in the *COX4I1* gene in a 3-year-old female patient suffering from poor growth, short stature, mild dysmorphic features, and bone marrow dysfunction. Pathogenicity was verified by complementation with the wild-type gene in the patient’s fibroblasts [9].

*COX4I1* is a nuclear gene encoding the common isoform of COX subunit 4 (COX4). COX4 has two isoforms: COX4 isoform 1 (COX4-1) and COX4 isoform 2 (COX4-2), encoded by the COX4I2 gene. COX4-1 is expressed under physiological conditions and is an essential structural and regulatory component of COX. COX4-2, the less common isoform, is primarily expressed in the lungs and at lower levels in the placenta, heart, brain, and pancreas. COX4-2 is preferentially expressed under hypoxia and other stress conditions [1,2,3,8]. Recently, we reported that the isoform switch may act as a compensatory mechanism occurring in COX4-1-deficient cells (in both knockdown fibroblasts and in the patient’s cells). We demonstrated the involvement of HIF-1α stabilization and nuclear translocation, which was followed by a COX4 isoform switch under normoxic conditions. We thus hypothesized that the patient’s relatively mild phenotype could be related to the isoform switch, which preserved the partial COX activity [10]. 

Our patient presented with clinical manifestations similar to Fanconi anemia. This syndrome is characterized by bone marrow failure, genomic instability, and predisposition to cancer. The “classical” Fanconi anemia (FA) syndrome results from mutations in genes participating in the FA pathway. This cellular process is activated when DNA replication is blocked due to DNA damage. The activation of the FA pathway in intact cells eventually leads to DNA repair. Considering the patient’s clinical phenotype, we also studied the contribution of COX deficiency to nuclear genomic stability. To this end, we demonstrated the occurrence of elevated nuclear double-stranded break (DSB) foci in COX4-1-deficient cells and found that the occurrence of these DSB foci was independent of ROS and ATP levels but dependent on the replicative state. We suggested that the pathomechanism underlying COX deficiency is associated with nuclear genomic instability and is independent of oxidative stress or energy depletion [11,12]. Conversely, oxidative stress associated with mitochondrial dysfunction has been implied previously in the classical form of Fanconi anemia in patients’ cells harboring mutations in the FA pathway genes [13,14]. Mitochondrial dysfunction also compels FA cells towards glycolytic metabolism [15], and this is similar to our observations in COX4-1-deficient cells [10].

To the best of our knowledge, the contribution of deficient mitochondrial respiration to genomic instability and its consequences has only been studied in the context of oxidative stress. In this study, we aimed to resolve the pathophysiological mechanism of COX4-1 deficiency with respect to its impact on nuclear genomic instability and thereby on potential clinical consequences. Our current study demonstrated that downregulated COX4-1 results in replicative stress and impaired nuclear DNA damage response. 

## 2. Results

Previously, we showed that restrictive replication conditions (glucose-free and serum-free) lead to reduced levels of DSB in COX4-1-deficient cells [12]. This evidence led us to hypothesize that COX4-1 deficiency would also reduce proliferation under permissive conditions (high-glucose media) in order to circumvent replicative stress and DSB. We also evaluated DSBs under PARP inhibition and observed a major significant elevation in DSB levels relative to untreated COX4-1-deficient cells, while the controls showed only a minor elevation. PARP inhibitors act by blocking the DNA repair pathways in which PARP participates (single-stranded break; base excision repair) resulting in the accumulation of single-stranded DNA breaks, which subsequently leads to DSB. In cells with an intact DNA damage response (DDR), these breaks will be mended through double-stranded repair pathways (homologous recombination; HR or nonhomologous DNA end joining; NHEJ). Accordingly, cells with impaired HR, for example those harboring pathogenic mutations in breast cancer genes BRCA1 or BRCA2, are hypersensitive to PARP inhibitors and persistently accumulate DSB due to the error-prone NHEJ [16]. Since we previously showed that COX4-1-deficient cells are hypersensitive to PARP inhibitor treatment, we assumed that the HR mechanism in COX4-1 deficiency could be affected as well. 

Consequently, we set out to examine DDR and proliferation in the patient’s cells harboring the K101N mutation and in COX4I1 knocked-down cells.

### 2.1. Nuclear DNA Instability Coincides with Proliferation

**Figure 1:**
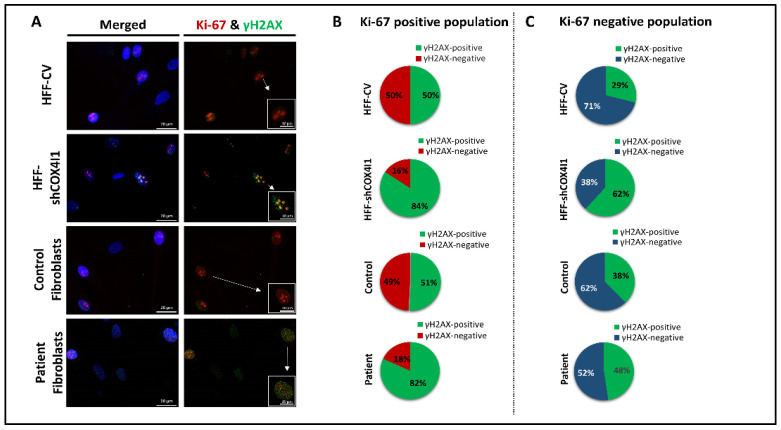
High levels of DSB coincide with Ki-67-positive cells in COX4-1-deficient cells. Both COX4-1-deficient cells (HFF-shCOX4I1 and patient) and their corresponding controls (HFF-CV and healthy control) from similar passages were seeded in equal amounts overnight on tissue culture slides and were fixed and stained simultaneously for γH2AX (green) and Ki-67 (red). Nuclei were visualized by Hoechst-3334 (blue) (**A**). The pie charts represent the distribution of DSBs (γH2AX, green) in proliferative (Ki-67-positive (**B**)) and in nonproliferative cells (Ki-67-negative (**C**)). At least 100 nuclei of each group of cells were analyzed and quantified. Increased colocalization of Ki-67 and γH2AX was observed in both COX4-1-deficient cells relative to corresponding controls.

We previously showed that COX4-1-deficient cells display high levels of DSB relative to controls by performing phospho-histone H2AX Ser139 (γH2AX) foci staining [11,12]. We also noted that cells maintained in glucose-free restrictive medium (where cells with impaired OXPHOS cease to proliferate) lacked DSB [12]. We therefore set out to determine whether elevated DSB correlates with the proliferative status seen in COX4-1 deficiency, by co-staining with both γH2AX and Ki-67 (Figure 1). Although a positive co-staining of both markers was observed in many cells, COX4-1-deficient cells displayed a higher level of co-staining (Figure 1A) The pie charts (Figure 1B,C) represent the distribution of both proliferative (Ki-67-positive) and nonproliferative (Ki-67-negative) cell populations stained for DSBs (γH2AX-positive or -negative). We observed a statistically significant (*p* < 0.05) positive correlation between the fraction of proliferating Ki-67 HFF-shCOX4I1 and patient’s cells that were also γH2AX-positive (84% and 82%, respectively), while the proliferative fraction of control cells had a lower tendency (≈50% γH2AX-positive) (Figure 1B). As expected, the nonproliferative fraction disclosed a decreased occurrence of DSBs in all cells (Figure 1C). The difference between proliferative and nonproliferative γH2AX-positive cells was most marked (82% vs. 48%) in the patient’s cells. These results are consistent with replicative stress, as replicating cells disclose more nuclear DNA damage than nonreplicating cells. 

### 2.2. CEL-Seq2 Analysis Identified DNA Repair as One of the Main Downregulated Pathways in COX4-1 Knockdown Cells 

**Figure 2:**
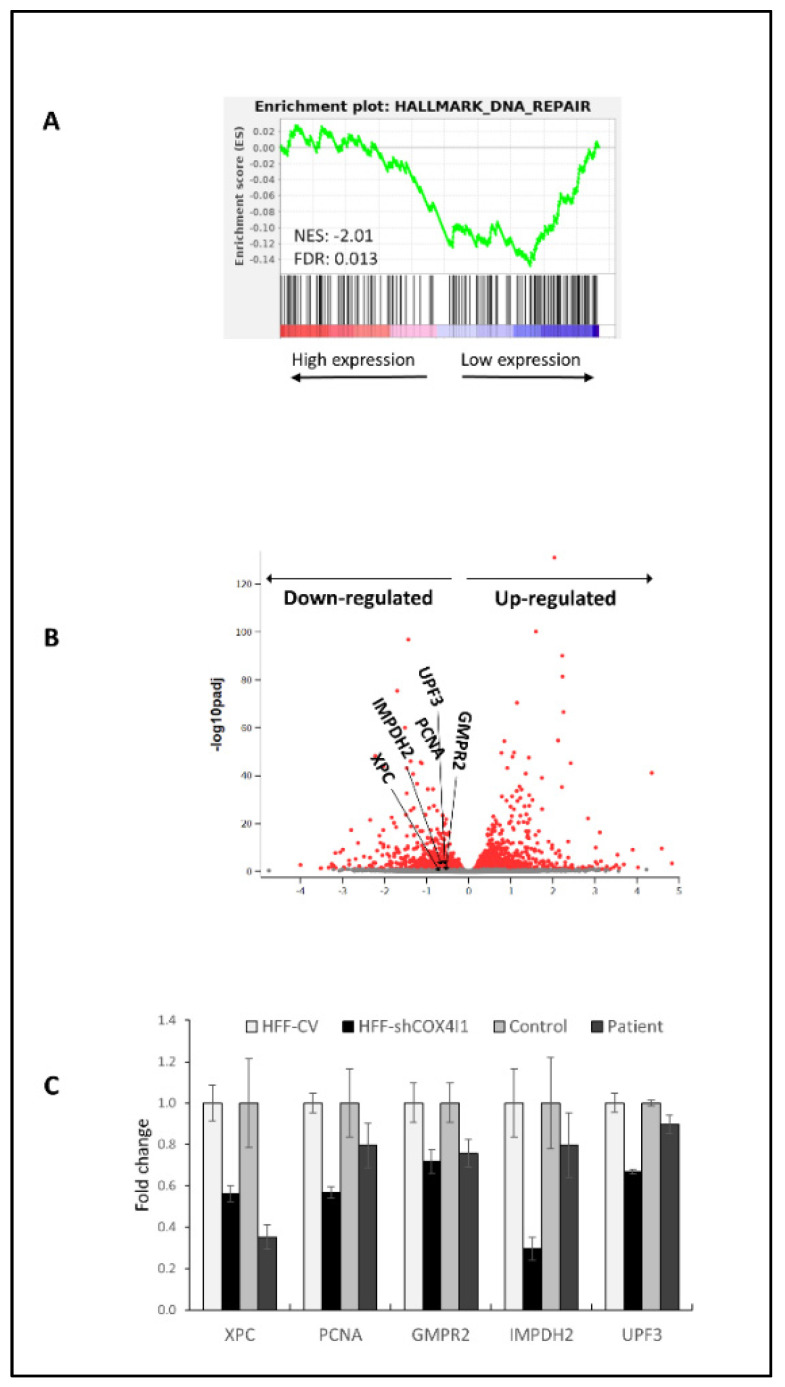
CEL-Seq2 analysis identifies DNA repair as one of the top downregulated pathways in COX4-1-deficient cells. Total RNA was isolated from human foreskin fibroblast cell line (HFF) and transfected with a control vector (HFF-CV) or HFF with downregulated COX4I1 (HFF-shCOX4i1). A HiSeq assay CEL-Seq2 analysis was performed and analyzed with the DESeq2 package. Significance threshold was set as FDR < 0.1. Downregulation of DNA repair was detected by gene set enrichment analysis (GSEA). NES: normalized enrichment signal; FDR: false discovery rate (**A**). In the volcano plot, each dot represents a gene (**B**). The x-axis indicates the log2 (fold change) of the expression of HFF-shCOX4I1 relative to healthy control fibroblasts, and the y-axis reflects −log10 of the FDR-adjusted *p*-value of this comparison. The colored dots pass the threshold for FDR. (**C**) Selected DNA repair genes in the volcano plot (XPC, PCNA, GMPR2, IMPDH2, UPF3) were validated by RT-qPCR in both COX4-1-deficient cells (HFF-shCOX4i1 and patient) and their corresponding controls. Values of RT-qPCR validation are presented as the log2 (fold change) in ±SD of biological triplicates.

Our previous and present results show that COX4-1-deficient cells display high levels of DSB foci, which are correlated with their proliferative status. Thus, we hypothesized that the observed senescence is an attempt to ameliorate DNA damage due to replicative stress, possibly due to impaired DNA damage response (DDR) [11]. To explore this possibility, we re-evaluated the previously performed CEL-Seq2 analysis of HFF-shCOX4I, which showed the upregulation of the glycolytic and hypoxia-related pathways in accordance with our bioenergetic studies [10]. In the current study, we noticed a significant downregulated DNA repair pathway, which presented an enrichment of several genes linked to DNA repair (the top 20% downregulated genes are listed in Supplementary Material Table S1 and depicted in Figure 2A,B). We verified five of the downregulated genes present in the DNA repair enrichment gene set (XPC, PCNA, GMPR2, IMPDH2, UPF3) in COX4-1-deficient cells by RT-qPCR (Figure 2C). These genes are marked in the volcano plot, Figure 2B. We also detected a qualitative but not quantitative correlation between HFF-shCOX4I1 and the patient’s cells (Figure 2C). This could possibly be attributed to different nuclear backgrounds. Nevertheless, a decreased expression in all five genes quantified was observed, which was in agreement with the CEL-Seq2 analysis. 

### 2.3. Impaired DNA Damage Response in COX4-1-Deficient Cells

**Figure 3:**
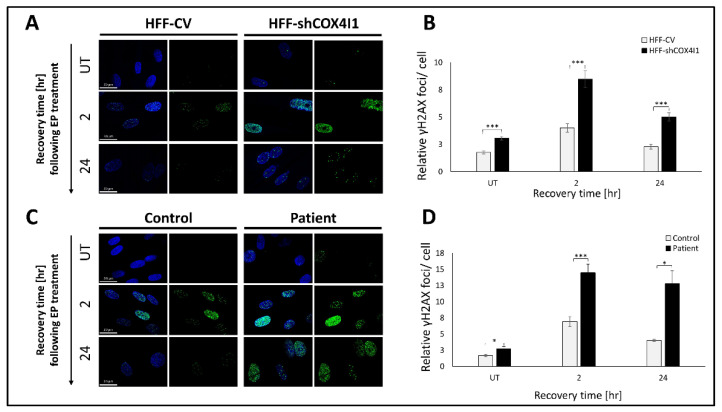
DNA damage recovery following etoposide (EP) treatment is less efficient in COX4-1-deficient cells. (**A**,**C**) Both COX4-1-deficient cells (HFF-shCOX4I1 and patient) and their corresponding controls (HFF-CV and healthy control) from similar passages were seeded in equal numbers overnight on tissue culture slides and, the following day, were exposed for 1 h to 100 µM etoposide (EP). Following 2 or 24 h of recovery, the cells were stained for DSBs anti-γH2AX antibody (green). Nuclei were visualized by Hoechst-3334 (blue), and the quantity of DSB foci per nucleus was analyzed and compared to untreated cells (UT) and respective controls. The micrographs were quantified and depicted as histograms (**B**,**D**) that show the γH2AX foci number per nucleus relative to t = 0 (meaning untreated cells) ± SEM of at least 100 nuclei, * *p* < 0.05, *** *p* < 0.001. The results suggest that both COX4I1-deficient cells showed impaired ability to respond and thereby to recover from DNA damage, while the controls exhibited an almost complete recovery 24 h after the exposure.

The present expression analysis, as well as our previous results showing increased sensitivity to PARP inhibitors [12], led us to hypothesize that the nuclear DNA repair system and, specifically, homologous recombination (HR) is less effective in COX4-1 deficiency. To address this question, we examined the ability of COX4-1-deficient cells (both HFF-shCOX4I1 and the patient’s cells) to recover from an external DNA insult, i.e., their ability to repair DNA damage caused by a short exposure to a DNA-damaging chemical (etoposide; EP). To that end, we quantified γH2AX foci at two different time points, after an hour of exposure to EP. As shown in Figure 3, both HFF-shCOX4I1 (Figure 3A,C) and the patient’s cells (Figure 3B,D) displayed an impaired ability to respond and fully recover from a genotoxic insult, while the damage in the corresponding controls was almost fully-repaired after 24 h of recovery. However, exposure to EP treatment induced an initial increase in γH2AX foci after 2 h of “recovery”. After 24 h of recovery, both HFF-shCOX4I1 and the patient’s cells displayed a significant prolongation of the γH2AX foci signal, while both control cells had almost comparable levels relative to the untreated cells. This result indicates that the increased levels of DSB observed in COX4-1-deficient cells can most probably be attributed to an impaired DNA damage response.

**Figure 4:**
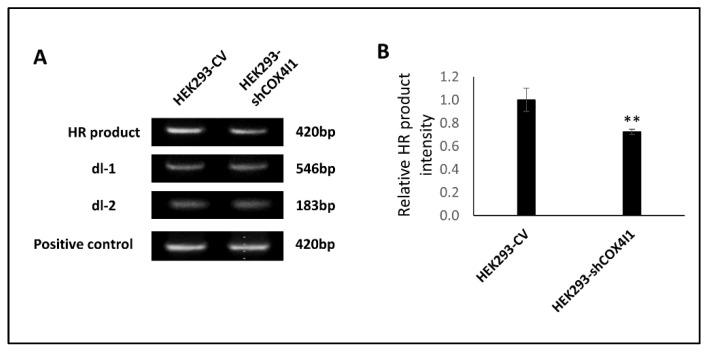
Downregulation of COX4-1 interferes with homologous recombination (HR) repair. Homologous recombination (HR) assay was performed on HEK293 cell line with either downregulation of COX4I1 (HEK293-shCOX4I1) or control (HEK293-CV) by transfecting with two mutated assay plasmids (dl-1 and dl-2) or a positive control plasmid according to the manufacturer’s instructions. DNA was isolated 24 h later and PCR reaction was performed to determine HR efficiency. A weaker intensity of the HR product was observed in the HEK293-shCOX4I1 relative to that of the HEK293-CV ((**A**), depicting a representative experiment out of three). When normalizing the intensities of the HR product to that of the controls of each type of cell (dl-1 and dl-2), a significantly decreased HR product was observed in the COX4-1-deficient cells relative to that of the corresponding control (**B**). Values are normalized to the mean value of the control ± SEM; ** *p* < 0.01, *n* = 3.

In an attempt to further elucidate the mechanism, we specifically examined HR efficiency by employing a commercially available homologous recombination assay, which requires the co-transformation of cells with two plasmids encoding the LacZα gene but with two different mutations. Only a functioning HR is able to restore the intact gene. As transformation is much less efficient in primary cells such as HFF, we constitutively knocked-down COX4I1 in the HEK293 cell line (HEK293-shCOX4I1) instead. The knockdown efficiency and the presence of DSB were both validated in the HEK293 cells before commencing, and the results were comparable to those we reported in HFF-shCOX4I1 cells [10] (Supplementary Material Figure S1). Subsequently, we co-transfected the mutant LacZα plasmids (dl-1 and dl-2) into both HEK293-shCOX4I1 and HEK293-CV overnight and analyzed the resulting products by PCR (Figure 4), detecting a 28% significant decrease in the HR efficiency compared to the control. Although the decrease was moderate, it was statistically significant and thus in accord with impaired DDR. 

### 2.4. Impaired Proliferation of COX4-1-Deficient Cells

We noted that COX4-1-deficient cells (patient fibroblasts and knockdown cells; HFF-shCOX4I1) proliferated relatively slowly compared to the respective controls of the same passages. This was especially true for the shCOX4I1 cells, which did not survive more than three to four passages. The slow proliferation was not attributed to apoptosis, as we detected no difference by TUNEL staining (results not shown). The preliminary results (Supplementary Material Figure S2) showed elevated β-galactosidase (SA-β-Gal) staining, which is associated with senescence [17]. Additionally, we estimated the averaged telomeric lengths of both HFF-shCOX4I1 and HFF-CV, as telomere attrition is an additional marker for senescence. Supplementary Material Figure S3A represents the averaged telomeric length of the knockdown cells relative to the that of the control cells (same nuclear background and passage). We observed a marked (≈2 Kb) reduction in the average telomeric lengths of HFF-shCOX4I1 cells relative to HFF-CV. We simultaneously assayed mitochondrial DNA content, which was relatively elevated (Supplementary Material Figure S2B). Regretfully, despite several attempts being made, we were not able to verify the occurrence of premature senescence by staining p16 in immunostaining or *CDKN2A* transcripts by quantitative reverse transcription polymerase chain reaction (RT-qPCR), although CEL-Seq2 analysis showed a 1.4-fold increase in expression. As for now, we cannot define the primary cause of impaired proliferation. Most likely, it can be attributed to DNA instability, but it could also be related to energy deficits, although the experiments were performed in high-glucose medium, which is permissive for OXPHOS-deficient cells. 

## 3. Discussion and Conclusions

### 3.1. Discussion

We demonstrated that COX4-1 deficiency coincides with accumulative DNA damage, primarily in Ki-67-positive cells (Figure 1), which is in accord with replicative stress. This is also compatible with FA pathway deficits, which are characterized by accumulative DNA damage, replication crisis, and chromosomal aberrations due to impaired DNA damage response (DDR) [18]. To strengthen this hypothesis, we proceeded to examine DDR by expression analysis (Figure 2) and experimentally demonstrated that COX4-1-deficient cells indeed displayed an impaired ability to respond and recover from a short exposure to a genotoxic agent along with a reduction in HR efficiency (Figure 3 and Figure 4). Notably, the present data are compatible to our previous results showing that the patient’s fibroblasts are hypersensitive to PARP inhibitors [12]. 

The reduced proliferation observed in COX4-1-deficient cells could be related to premature senescence, which occurs in response to the persistent nuclear DNA damage that we have previously reported. To this end, we demonstrated elevated SA-β-Gal in both COX4-1-deficient and control cells at three sequential passages (Figure 4) and also by telomere shortening (Supplementary Material Figure S3), while ruling out apoptosis. However, we failed to confirm premature senescence by other means, and thus do not rule out that impaired proliferation could be attributed to factors other than chromosomal instability and senescence. 

Similarly to our observations, FA patients’ cells have demonstrated cellular features related to senescence, including decreased proliferation, a short lifespan of fibroblasts, and the increased expression of SA-β-gal. Telomere shortening is another feature of senescence, and DNA breakage and is also associated with FA, which fits well with our present findings. Although the telomere shorting in our HFF-shCOX4I1 in vitro cell system was less prominent than in blood cells from common FA complementation groups, it was markedly evident. Indeed, recently, FA has been redefined as an accelerated aging (AA) disease (including premature aging syndromes and aging-associated diseases) [19,20,21]. Observing the clinical and cellular manifestations, it seems that COX4-1 deficiency and other mitochondrial diseases could possibly belong to this group as well. The observed increase in mtDNA content was most probably a result of some compensatory mechanism we have previously observed in COX-deficient cells [22].

Notably, this in vitro investigation was initiated by the phenotype that our patient initially presented at the age of 2 years, although the COX4I1 mutation was only identified and reported when she was 5 years old (9). Recent evaluation at the age of 9 years showed that she still presents with severe growth faltering (failure to thrive: weight of 12 kg (significantly less than −2 SD); height 80 cm (significantly less than −2 SD); and occipitofrontal circumference of 49 cm (at just −2 SD)). Nevertheless, she has reasonable school performance and adequate mental and psychosocial development. To the best of our knowledge, she did not develop any seizure disorder or any other abnormal neurological signs or symptoms, including malignancy. Her last blood counts showed mild normocytic anemia, with normal WBC and platelet counts (Hb = 11.3 gm/dL; Hct = 32.8%; MCV = 87.2 FL; BC = 3.76 × 10^6^/µL; WBC = 7.4 × 10^3^/µL; PLT = 438 × 10^3^/µL). She is maintained only on a daily dose of 100 mg vitamin C (which was initiated based on our previous studies in fibroblasts [23]). Interestingly, our in vitro findings recapitulate the stunted growth in the presence of chromosomal instability, but without evidence of malignant transformation in the patient.

We are aware of the limitations to this in vitro study. First, we could not exclude the contribution of different nuclear and mitochondrial genomic backgrounds while examining the patient’s fibroblasts and, moreover, age-matched healthy controls were (for obvious reasons) not available. Still, generating the knockdown vs. control from the same cell line enabled us to overcome this issue. Second, the patient’s cells, especially the HFF-shCOX4I1 cells, were defective in proliferation, and the number of passages was very limited and prevented further in-depth studies. Thus, we had to use the HR assay on the cancerous cell line HEK293, which was far from an ideal system. Third, in this study we did not provide a direct link between impaired nuclear DDR and the COX. We speculated that this could be linked to *Higd1a*, a mitochondrial protein which serves as a positive regulator of COX and has very recently been shown to translocate to the nucleus upon DNA damage, where it regulates homologous recombination [24,25]. This issue will be addressed in the future, in the context of exciting evolving field research aimed at unravelling the interplay between mitochondria and nuclei reviewed in [26]. Despite the shortcomings of this investigation, we can confirm the presence of replicative stress and, to some extent, premature senescence in COX4-1 deficiency. We cannot exclude that these features could be a part of the pathomechanism of other primary mitochondrial diseases as well. 

### 3.2. Conclusions

Taken together, we conclude that the observed nuclear DNA instability in COX4-1 deficiency is due to replicative stress linked to impaired nuclear DNA damage response and is most probably a contributing factor to impaired cell growth. This may explain some of the clinical features observed in in the patient, including chromosomal instability and stunted growth.

## 4. Materials and Methods 

### 4.1. Tissue Cultures

Previously established skin primary fibroblast cultures from the patient (with informed consent; all experimental protocols were approved by Hadassah Medical Center IRB #0485-09 and all methods were carried out in accordance with relevant guidelines and regulations) (7); Human foreskin fibroblasts (HFF-1) (ATCC, Manassas, VA, USA); and HEK293 (Invitrogen, Carlsbad, CA, USA) cell lines were maintained in high-glucose DMEM supplemented with 15% fetal bovine serum, L-glutamine, pyruvate, and 50 µg/mL uridine (Biological Industries, Beit Ha’emek, Israel). For immunocytochemistry, cells were seeded on u-slide 8-well ibiTreat sterile tissue culture slides (NBT; New Biotechnology Ltd., Jerusalem, Israel). For RNA analysis, cells were grown in 6-well plates. For DSB staining and proliferation assays, cells were seeded on u-slide 8-well ibiTreat sterile tissue culture slides. All cells were incubated at 37 °C in an atmosphere of 5% CO_2_ and 19.3% O_2_ (considering the atmospheric pressure at 750 m elevation). Cells from passages 7 or lower were used for the experiments. Viability was assessed by trypan blue staining. 

### 4.2. RNA Interference 

We employed the MISSION^®^ shRNA plasmid DNA vector system shRNA #TRCN0000232554 to constitutively knockdown the expression of COX4I1, as we have previously described (7). A nonmammalian shRNA Control Plasmid DNA target served as a control vector (Sigma-Aldrich-Merck, Darmstadt, Germany). Briefly, we introduced each of the DNA plasmids into HFF-1 or HEK293 cells by co-transfection with pLP1, pLP2, and pLP/VSVG plasmids using lipofectamine (ViraPower; Invitrogen, Carlsbad CA, USA). Human foreskin fibroblasts and HEK293 cells were infected with viral supernatant containing polybrene. Stably transfected cells were selected with puromycin (2 µg/mL) for three weeks. For experiments, the cells were maintained in permissive medium without puromycin. Knockdown was verified by RT-qPCR, as we have previously described [10].

### 4.3. Immunofluorescence Staining

Fifteen thousand cells per well were seeded on u-slide 8-well ibiTreat sterile tissue culture slides (ibidi GmbH, Gräfelfing, Germany). On the following day, the cells were fixed with 4% formaldehyde for 10 min at room temperature, and then permeabilized with ice-cold 90% methanol for an additional 10 min at 4 °C. After blocking with 1% BSA/PBS for 30 min at room temperature, slides were incubated with primary antibody for 1 h at room temperature. The cells were washed five times with PBS containing 0.05% Tween-20, and then were incubated with secondary antibody for 1 h at room temperature in the dark. The following primary antibodies were used for immunofluorescence: Ki-67 (1:250; Cat#: ab16667, Abcam, Cambridge, UK). Secondary antibodies: anti-Rabbit Cy5 (Cat#: 711-175-152) and anti-mouse DyLight 488 (Cat#: 115-485-062) (both from Jackson Immuno Research, Laboratories, Baltimore Pike, PA, USA). The slides were subsequently washed five times with PBS and nuclei were stained with Hoechst 33342, NucBlue live cell stain (Molecular probes, Life Technologies, Eugene, OR, USA). The cells were examined by fluorescence confocal microscopy, ×40 magnifications (Nikon A1R) (for analysis). Image analyses were performed by the quantification of γH2AX foci per nucleus using the Image J software http://imagej.nih.gov/ij (last accessed on 5 October 2021) (National Institute of Health, Bethesda, MD, USA).

### 4.4. Nuclear DNA Double-Stranded Breaks (DSB)

Fifteen thousand cells per well were seeded on u-slide 8-well ibiTreat sterile tissue culture slides (ibidi GmbH, Gräfelfing, Germany) in each of the following experiments. For nuclear DSB foci evaluation, cells were seeded and incubated overnight; on the following day, the cells were fixed, permeabilized, blocked, and incubated with antibodies against phosphor-histone γH2AX Ser139 using the Oxiselect DNA Double Stranded Break Staining Kit according to the manufacturers’ instructions (Cell Biolabs Inc., San-Diego, CA, USA). For DNA damage recovery experiments, cells were seeded and incubated overnight and, on following day, the cells were incubated with 100 μM of etoposide (EP) for 1 h; subsequently, the medium was replaced with a fresh high-glucose medium, and the cells were incubated for 2 or 24 h at 37 °C, 5% CO_2_. Following 2 or 24 h of recovery, nuclear DSBs were determined using the Oxiselect DNA Double Stranded Break Staining kit. Nuclei were stained with Hoechst 33342, NucBlue live cell stain (Molecular probes, Life Technologies, Eugene, OR, USA). Preparates were examined by fluorescence confocal microscopy with ×60 magnification (Nikon A1R), and the number of nuclei with DSB was estimated by observing at least a hundred nuclei and calculating the relative number of γH2AX foci per nucleus using the Image J software https://imagej.nih.gov/ij/ (last accessed on 5 October 2021). For double staining experiments, cells were seeded and incubated overnight and, on following day, the cells were fixed, permeabilized, blocked (according to the manufacturer’s instructions), and incubated simultaneously for 1 h with both antibodies (1:100 for γH2AX, 1:250 for Ki-67); subsequently, cells were incubated with a mix of two secondary antibodies (FITC and Cy-5, respectively), washed, and stained with NucBlue. 

### 4.5. mRNA Expression by Linear Amplification and Sequencing—CEL-Seq

Total RNA was extracted on 3-5 separate occasions from HFF-shCOX4I1 and HFF-CV using Tri-Reagent (Sigma-Aldrich-Merck, Darmstadt, Germany) according to the manufacturer’s instructions. Qualification and quantification were measured using Qbit. Hi-seq assay using CEL-Seq approach was performed at the Technion Genome Center, Haifa, Israel. Statistical and bioinformatics analyses were performed in collaboration with the Bioinformatics unit of the Hebrew University of Jerusalem, Faculty of Medicine. Differential expression analysis was performed with the DESeq2 package (v1.22.1) [27]. In order to identify altered biological functions between HFF-shCOX4I1 and HFF, we ran gene set enrichment analysis (GSEA, reference: https://www.ncbi.nlm.nih.gov/pmc/articles/PMC1239896/) (last accessed on 20 February 2021). GSEA uses whole differential expression data (cut-off-independent) to determine whether a priori defined sets of genes show statistically significant, concordant differences between two biological states. We used the hallmark gene set collection from the molecular signatures database (MsigDB). The expression data results (GSE166429) are available on the GEO (Gene Expression Omnibus) website (https://www.ncbi.nlm.nih.gov/geo/; accessed on 20 February 2021).

### 4.6. Quantitative Reverse Transcription Polymerase Chain Reaction (RT-qPCR)

Total RNA was extracted using Tri-Reagent (Sigma-Aldrich-Merck, Darmstadt, Germany), according to the manufacturer’s instructions, from HFF-shCOX4I1, HFF-CV, patient, and healthy control cells. Then, cDNA from poly(A) + mRNA was generated using Improm II (Promega, Madison, WI, USA). Real-time, quantitative PCR for the quantification of XPC, PCNA, GMPR2, IMPDH2, UPF3B, COX4I1, COX4I2, GUSB, and GAPDH transcripts was performed using Fast SYBR GreenMaster Mix and the ABI PRISM7900HT sequence detection system (Applied Biosystems, Foster City, CA, USA). Primer sequences used for qPCR are supplied in the Supplementary Material. 

### 4.7. Homologous Recombination Assay

A homologous recombination assay was performed according to the manufacturer’s instructions (Norgen Biotek Corp., Thorold, ON, Canada, cat #35600). In brief, HEK293 cells expressing either control vector (CV) or shCOX4I1 were co-transfected with both dl-1 and dl-2 plasmids or a positive control plasmid (to assess transfection efficiency), and total genomic DNA was isolated 24 h later using a DNeasy^®^ Blood & Tissue Kit (catalog No. 69504 and 69506; Qiagen, Hilden, Germany). PCR reaction was performed using the supplied primers to determine HR efficiency and with the manufacturer’s recommendation—2X PCR Master Mix (Norgen Biotek Cat# 28007). If HR in COX4-1-deficient cells is perturbed, the dl-1 and dl-2 plasmids will recombine less subsequently, producing less of the PCR product compared to control cells; therefore, the amount of PCR product is directly associated with HR efficiency. The amount of recombinant product for each reaction was calculated by comparing the intensity of the HR product to the intensities observed in the control (dl-1 and dl-2). Analysis of PCR products was performed using the Image J software https://imagej.nih.gov/ij/ (last accessed on 5 October 2021).

### 4.8. Senescence, Telomere Length, and Apoptosis Detection 

Senescence-associated β-galactosidase (SA-β-gal) staining was performed using the Senescence β-Galactosidase Staining Kit (MBL International Crop., Woburn, MA, USA, cat #JM-K320-250) according to the manufacturer’s protocol. The same number (15,000) of cells from the same passage were seeded on u-slide 8-well ibiTreat slides. Following 48 h, cells were washed with PBS X1 and fixed with the supplied fixing solution for 10 min at RT. Next, the cells were washed once in PBS to remove the fixing solution and incubated with a freshly prepared staining solution mix containing the X-gal substrate at 37 °C overnight. Afterward, SA-β-gal-positive cells (senescent cells) were identified as blue-stained cells under ×20 magnification using a Nikon-TI fluorescence microscope. To quantify SA-beta-gal activity, the percentage of positively stained blue cells versus total cells was calculated. For each type of cell, at least 120 cells were counted. For estimating telomere length, cells were seeded in triplicated in a 6-well plate. Total genomic DNA was isolated 24 h later using a DNeasy^®^ Blood & Tissue Kit (catalog No. 69504 and 69506; Qiagen), and telomeric lengths were quantified by RTqPCR (Absolute Human Telomere Length and Mitochondrial DNA Copy Number Dual Quantification qPCR Assay Kit, ScienCell, Carlsbad, CA, USA; Cat# 8958) according to the manufacturer’s instructions. The results were compared to reference genomic DNA containing a 100-base pair (bp) telomere sequence, and average telomere length was calculated following the manufacturer’s instructions. Apoptosis was estimated by terminal deoxynucleotidyl transferase (TdT)-mediated deoxyuridine triphosphate (dUTP) nick-end labeling (TUNEL) using the in situ cell-death assay kit (Roche Diagnostics GmbH, Mannheim, Germany) according to the manufacturer’s instructions. The nuclei of the apoptotic cells containing DNA strand breaks were stained green (TUNEL apoptosis signal), and the overall nuclei were stained with Hoechst 33342 and examined by fluorescence confocal microscopy, ×40 magnifications (Nikon A1R) (for analysis). Image analyses were performed by ImageJ software, whereby we analyzed the green fluorescence signals (TUNEL apoptosis) per nucleus. Results were represented as the ratio between green (TUNEL signal) and blue (nucleus) intensities.

### 4.9. Statistical Analysis

All experiments were performed in triplicates on at least two different occasions. Statistical analysis was carried out by two-tailed Student’s unpaired *t*-test using IBM SPSS Statistics for Windows, version 24.0. (IBM Corp. Armonk, NY, USA); *p* values < 0.05 were considered statistically significant.

## Data Availability

The expression data results (GSE166429) are available on the GEO (Gene Expression Omnibus) website (https://www.ncbi.nlm.nih.gov/geo/) (last accessed on 20 February 2021).

## Supplementary Materials

The following supporting information can be downloaded at: www.mdpi.com/article/10.3390/ijms23084149/s1.
