# Supplementary material for: Replicative Stress Coincides with Impaired Nuclear DNA Damage Response in COX4-1 Deficiency
Source: Int J Mol Sci. 2022 Apr 8;23(8):4149. doi: 10.3390/ijms23084149 (PMC9029573; doi:10.3390/ijms23084149)
Supplement: Supplementary file 1 [file ijms-23-04149-s001.zip › ijms-1664950-supplementary.pdf]

SUPPLEMENTARY MATERIAL

Table S1. List of ranked top 20% enriched genes linked to DNA repair

PROBE/GENE SYMBOL	ALIAS NAME	RANK METRIC SCORE
TYMS	Thymidylate Synthase	-5.48
TP53	Tumor Protein P53	-4.56
**IMPDH2**	**Inosine Monophosphate Dehydrogenase 2**	**-4.41**
**UPF3B**	**UPF3B Regulator Of Nonsense Mediated mRNA Decay**	**-4.14**
**GMPR2**	**Guanosine 5'-Monophosphate Oxidoreductase 2**	**-3.66**
NELFCD	Negative Elongation Factor Complex Member C/D	-2.96
**PCNA**	**Proliferating Cell Nuclear Antigen**	**-2.88**
**XPC**	**XPC Complex Subunit, DNA Damage Recognition and Repair**	**-2.75**
TAF9	TATA-Box Binding Protein Associated Factor 9	-2.61
NT5C	5', 3'-Nucleotidase, Cytosolic	-2.52
GTF3C5	General Transcription Factor IIIC Subunit 5	-2.5
SNAPC5	Small Nuclear RNA Activating Complex Polypeptide 5	-2.39
RFC3	Replication Factor C Subunit 3	-2.21
GSDME	Gasdermin E	-2.14
ERCC3	ERCC Excision Repair 3, TFIIH Core Complex Helicase Subunit	-2.13
RFC4	Replication Factor C Subunit 4	-2.11
TAF1C	TATA-Box Binding Protein Associated Factor, RNA Polymerase I Subunit	-2.08
ERCC4	ERCC Excision Repair 4, Endonuclease Catalytic Subunit	-2.06
ZNRD1	RNA Polymerase I Subunit H	-1.99
POLR2G	RNA Polymerase II Subunit G	-1.91
REV3L	REV3 Like, DNA Directed Polymerase Zeta Catalytic Subunit	-1.89
SAC3D1	SAC3 Domain Containing 1	-1.87
PDE4B	CAMP-Specific 3',5'-Cyclic Phosphodiesterase 4B	-1.8
HCLS1	Hematopoietic Cell-Specific Lyn Substrate 1	-1.69
VPS28	VPS28 Subunit Of ESCRT-I	-1.66
NFX1	Nuclear Transcription Factor, X-Box Binding 1	-1.6
DDB2	Damage Specific DNA Binding Protein 2	-1.5
AK3	Adenylate Kinase 3	-1.5
NCBP2	Nuclear Cap Binding Protein Subunit 2	-1.48

Expression of genes in bold were verified by RTqPCR

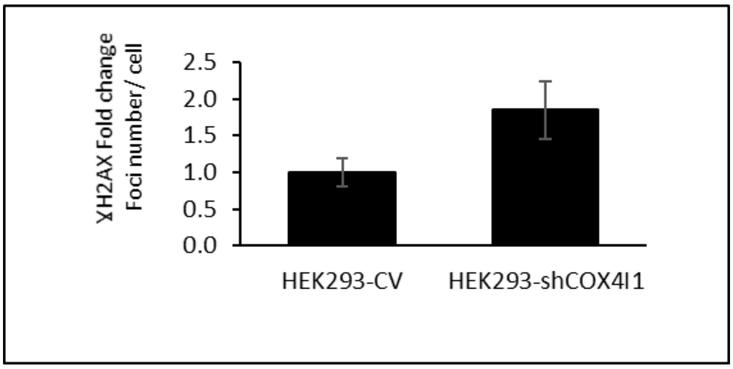

Figure S1 DSBs in HEK293 cells with downregulated COX*4I1*

HEK293 cells were transfected with a control vector (HEK293-CV) and HEK293 with downregulated *COX4I1* (HEK239-shCOC4I1), seeded on coverslips and stained double stranded DNA breaks with ɣH2AX antibodies. Nuclei were stained by NucBlue. At least 100 nuclei of each group of cells were analyzed and quantified ,mean ± SEM is depicted in the histogram .

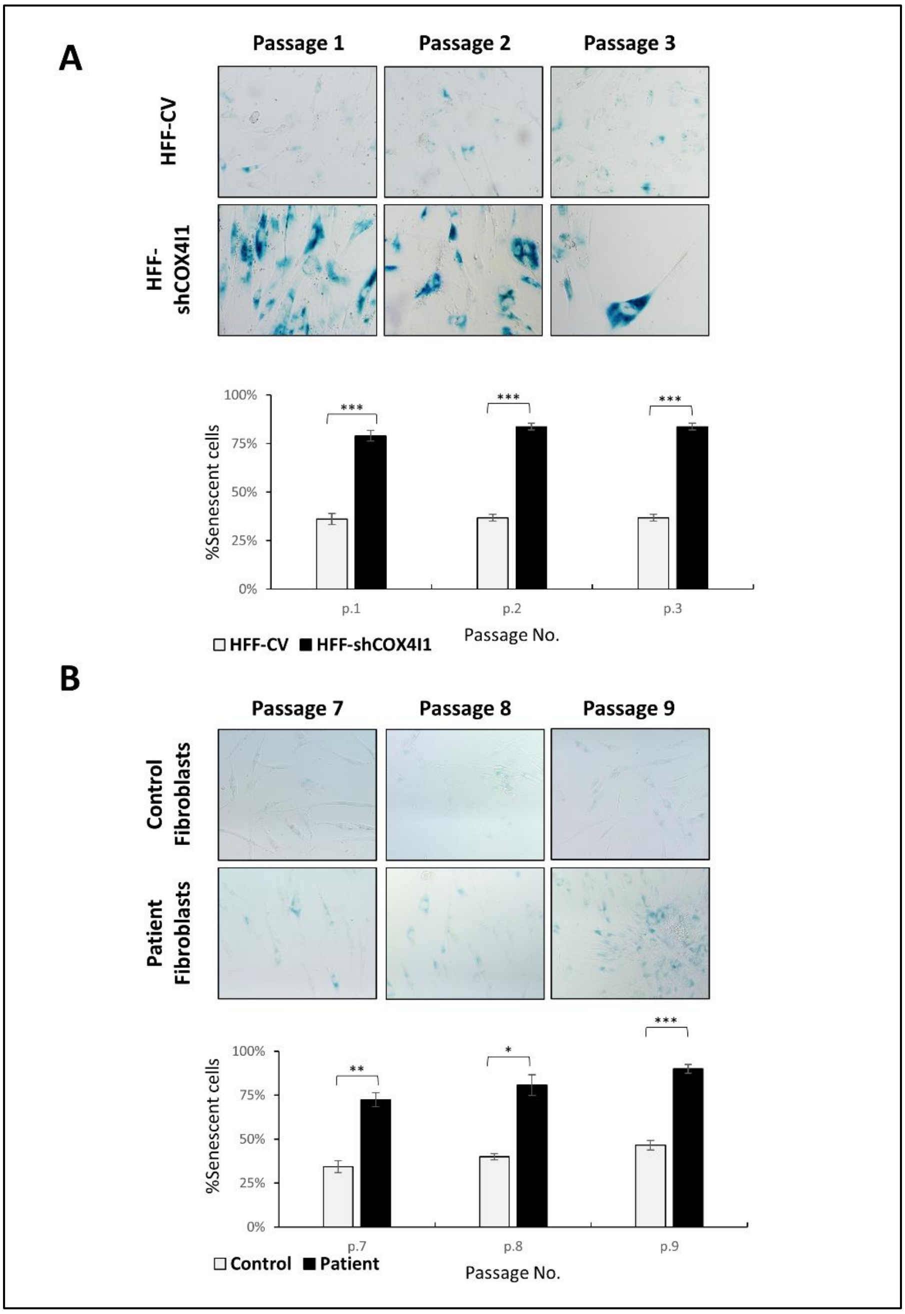

Figure S2. β-galactosidase staining. β-galactosidase (SA-β-gal) staining of COX4-1-deficient cells (HFF-shCOX4I1 (A) and patient (B)) and their corresponding controls (HFF-CV and healthy control). Representative micrographs from three shown constitutive passages (p) are shown (A,B). The percentage of positive (blue) cells was quantified and depicted in bar graphs under the micrographs, (mean ± SEM n>100, *p<0.05, **p<0.01, ***p<0.005) (A,B).

A B

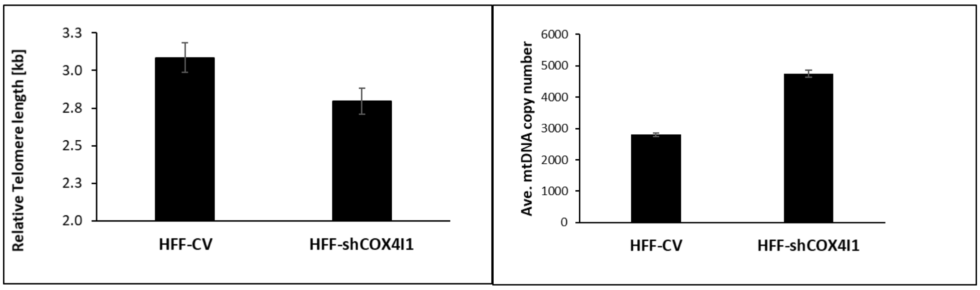

Figure S3 Telomere length. Telomere length (A) and mitochondrial DNA (mtDNA) (B) in was estimated in human foreskin fibroblast cell line (HFF) HFF transfected with a control vector (HFF-CV), HFF with downregulated COX4I1 (HFF-shCOX4I1) by RTqPCR, showing significantly decreased telomere length in HFF-shCOX4I1 cells, while mt DNA was elevated (mean ± SEM of triplicates)

Table S2. Primer sequences used for qPCR

<u>Gene</u>	<u>Forward primer</u>	<u>Reverse primer</u>
XPC (NM_004628)	5′-GATGACCTCAGGGACTTTCC-3′	5′-AAGACAGCAATGAAGAAGAGGA-3′
PCNA (NM_002592.2)	5′-AATTCCAGAACAGGAGTACAGC-3′	5′-GAGATGCTGTTGTAATTTCCTGTG-3′
GMPR2 (NM_001002002.3)	5′- CATCATGGCAGGGAATGT-3′	5′-AAAGTGGGAATTGGGCC-3′
IMPDH2 (NM_000884.3)	5′-ACAGGGATTCATCACAGACC-3′	5′-TGGAAGAGATAATGACAAAGAGG-3′
UPF3B (NM_080632.3)	5′-GGACTATCGATGATGATCCAGA-3′	5′-GAGACACTGCTAGAGGAAATAGA-3′
COX4I1 (NM_001861.6)	5′-TTTCACCGCGCTCGTTAT-3′	5′-CTTCATGTCCAGCATCCTCTT-3′
COX4I2 (NM_032609)	5′-GAAGACGAGGGATGCACAG-3′	5′-GGCTCTTCTGGCATGGG-3′
GUSB (NM_000181.4)	5′-GAAAATATGTGGTTGGAGAGCTCATT-3'	5′-CCGAGTGAAGATCCCCTTTTTA-3′

GAPDH (NM_001357943.2)	5'-CAAGAGCACAAGAGGAAGAGAG-3'	5′-CTACATGGCAACTGTGAGGAG-3′
